# The Morphology of Inner Cell Mass Is the Strongest Predictor of Live Birth After a Frozen-Thawed Single Embryo Transfer

**DOI:** 10.3389/fendo.2021.621221

**Published:** 2021-02-24

**Authors:** Jihui Ai, Lei Jin, Yu Zheng, Peiwen Yang, Bo Huang, Xiyuan Dong

**Affiliations:** Reproductive Medicine Center, Tongji Hospital of Tongji Medical College of Huazhong University of Science and Technology, Wuhan, China

**Keywords:** inner cell mass, single embryo transfer, live birth, pregnancy, miscarriage

## Abstract

**Background:**

The scoring system for human blastocysts is traditionally based on morphology; however, there are controversies on the effect of morphology parameters on pregnancy outcomes. The aim of this study is to evaluate the predicting value of each morphology parameter on pregnancy outcomes in a setting of single embryo transfer.

**Methods:**

This is a retrospective cohort study on patients undergoing frozen-thawed single blastocyst transfer at our center, between Jan. 2009 and Dec. 2018. A total of 10,482 cycles were analyzed. The blastocysts were scored according to the expansion and hatching status, morphology of inner cell mass (ICM), and cells of trophectoderm (TE). The primary outcome measure was live birth rate. One-way analysis of variance, chi-square test, and multiple logistic regression were used for statistical analysis.

**Results:**

The clinical pregnancy rate was lower in the blastocysts of stage 3 (48.15%), compared with those of stage 4 (56.15%), stage 5 (54.91%), and stage 6 (53.37%). The live birth rate was lower in the blastocysts of stage 3 (37.07%), compared with those of stage 4 (44.21%) and stage 5 (41.67%). The rates of clinical pregnancy (A: 66.60%, B: 53.25%, C: 39.33%) and live birth (A: 54.62%, B: 41.29%, C: 28.45%) were both decreased with decreasing grade of ICM morphology, and these differences were pairwise significant. The miscarriage rate of blastocysts with ICM grade A was lower, compared with ICM grade C (17.53 *vs.* 27.66%). Blastocysts with TE morphology of C had lower rates of clinical pregnancy (43.53%) and live birth (32.57%), compared with those with TE morphology of A and B (clinical pregnancy rate: 64.26% for A, 58.11% for B; live birth rate: 52.74% for A, 45.64% for B). There were no significant differences in rates of clinical pregnancy, live birth, and miscarriage between the blastocysts with TE grade A and B.

**Conclusions:**

The blastocyst expansion stage, ICM grade, and TE grade are all associated with pregnancy outcomes. ICM grade is the strongest predictor of live birth. A blastocyst with stage 4–5, ICM grade A, and TE grade A/B should be given priority for single embryo transfer.

## Introduction

*In Vitro* Fertilization (IVF) is an effective treatment for infertility, and has been widely used in the past decades. With the progress of embryo culture and vitrification, surplus blastocysts can be used by frozen-thawed embryo transfer (FET). In order to achieve a higher pregnancy rate, multiple embryos were routinely transferred; however, this practice resulted in multifetal pregnancies. Previous studies have reported increased risks of adverse maternal and perinatal consequences in multifetal pregnancies (1, 2). Therefore, single embryo transfer (SET) is advocated, aiming to promote singleton gestation and reduce the number of multiple pregnancies (3). Accordingly, there is a growing use of SET in China.

There are two basic techniques for cryopreservation of embryos: the slow freezing and vitrification. Vitrification has been demonstrated to result in a higher survival rate of embryos, and increased clinical pregnancy rate (CPR) and live birth rate (LBR) (4). With this progress in cryopreservation techniques, FET can yield similar or even better pregnancy and live birth rates compared with fresh transfer today (5, 6). It has been hypothesized that supra-physiological conditions in fresh cycles may affect endometrial receptivity, and impair the implantation. In contrast, FET provides a more favorable intrauterine environment for the attachment, implantation, and placentation of embryos. In addition, FET does not increase the risks of obstetric and neonatal complications (7).

Improved culture conditions allow embryos to be extendedly cultured to blastocyst stage. Blastocyst transfer improves pregnancy outcomes, and therefore has been widely used by many IVF centers. Previous studies comparing day 5 and day 6 blastocysts reported conflicting results. Some studies indicated comparable CPR and LBR between blastocysts of day 5 and day 6 (8, 9). However, more recent studies showed higher CPR and LBR after transfer with day 5 blastocysts (10, 11). It is the consensus that day 5 and 6 blastocysts have a better prognosis for pregnancy compared with delayed blastocysts (day 7 or 8) (12, 13). Embryo selection is crucial to achieving pregnancy. The increasing use of SET further highlights the need for an effective method in identifying the best embryos. In embryo assessment, the blastocyst scoring system based on morphology is traditionally used (14). Briefly, this method includes three morphological features: the blastocyst expansion and hatching stage, size and compactness of the inner cell mass (ICM), and cohesiveness and number of cells of the trophectoderm (TE). However, there is no consensus on the predicting value of each parameter. Furthermore, previous studies reported conflicting results on which parameter was the strongest predictor. Some studies showed that ICM grade had the best predicting effect (15–17), while the others indicated that expansion stage and TE grade were stronger predictors (18–20).

Since relatively late start of SET, it lacks clinical evidence on pregnancy outcomes after SET in Chinese population. Moreover, there is a need for further knowledge of the association between blastocyst morphology and pregnancy outcomes. The object of this study is to evaluate the effect of each morphology parameter on pregnancy outcomes after SET.

## Materials and Methods

### Study Population

This is a retrospective cohort study on patients undergoing FET at our center, between Jan. 2009 and Dec. 2018. All the cycles were transferred with a single frozen-thawed blastocyst. The cycles of patients with reproductive malformation, donation of oocytes, and preimplantation genetic testing (PGT) were excluded. This study was approved by the Institution Review Board (IRB) of Tongji Hospital. All patients gave the written informed consent for their clinical records to be used in research. Patient information was anonymized before analysis.

### Clinical Protocols

The down-regulation methods included the depot GnRH-a, daily GnRH-a, and GnRH-antagonist protocols. The ovarian stimulation was initiated with recombinant FSH (Gonal-F, Merck Serono, Switzerland) 112.5-225 IU subcutaneously (SC). The starting dose of recombinant FSH was based on the age, AFC, AMH, FSH, body mass index (BMI), and treatment history. Follicle development was monitored using transvaginal ultrasound scan, as well as serum estradiol (E2), progesterone, and LH level. Recombinant hCG (Ovidrel, Merck Serono, Switzerland) 0.25 mg SC was administered when at least two follicles reached a diameter of ≥18 mm. After 36–38 h, oocytes were retrieved.

### *In Vitro* Fertilization

Sequential culture media were used for oocyte and embryo culture. From 2009 to 2017, the oocytes were incubated in G-IVF medium (Vitrolife, Sweden). From 2018 onwards, both G-IVF medium and FM (Cook, Australia) were used. The oocytes were inseminated 3–4 h after retrieval. Fertilization was checked 16–18 h after insemination. The presence of two pronuclei was defined as normal fertilization. From 2009 to 2017, fertilized oocytes were continuously cultured in G1 medium (Vitrolife, Sweden) till day 3 of development. From 2018 onwards, CM (Cook, Australia) was also used. Morphological evaluation of the cleavage stage embryos was performed on day 2 and day 3 based on the number of blastomeres, the percentage of cytoplasmic fragmentation, the equality and multinucleation of blastomeres, and early compaction.

### Blastocyst Culture and Grading

From 2009 to 2017, G2 medium (Vitrolife, Sweden) was used for blastocyst culture. From 2018 onwards, both G2 medium and BM (Cook, Australia) were used. In 2009, the incubators were CO_2_ incubator C200 (Labotect, Germany) with control of CO_2_ (6%). From 2010 onward, they were replaced with the CO2 incubator C60 (Labotect, Germany) and K-MINC-1000 (Cook, Australia) with control of CO_2_ (6%), O_2_ (5%), and N_2_ (89%). Embryos were further cultured at 37°C till day 5 or 6, and very few blastocysts were cultured to day 7.

Blastocysts were scored by at least two experienced embryologists, using Gardner and Schoolcraft’s grading system (21). Mainly, three morphological parameters were evaluated: the expansion stage, ICM grade, and TE grade. In this study, expansion stage was graded into four degrees: 3, a full blastocyst with a blastocoele completely filling the embryo; 4, an expanded blastocyst with a blastocoele larger than that of the full blastocyst, and with a thinning zona pellucida; 5, a hatching blastocyst escaping from the zona pellucida; and 6, a hatched blastocyst that has completely escaped from the zona pellucida. ICM was graded into three degrees: A, many tightly packed cells; B, many loosely grouped cells; and C, very few cells. TE was graded into three degrees: A, many cells forming a cohesive epithelium; B, few cells forming a loose epithelium; and C, very few cells. To maintain standardized evaluation, following measures were employed: (1) Embryologists responsible for embryo grading were rigorously trained, qualified, and experienced. An identical atlas of blastocysts with different morphology parameters was provided to all the embryologists. In this study, six experienced embryologists scored the blastocysts. (2) At least two operators scored each blastocyst simultaneously. If it was difficult to give an identical score, a supervisor would provide his/her opinion. (3) Our center has obtained the ISO9001 certification for quality assessment. Blastocyst scoring was followed by ISO9001-Standard Operation Protocol (SOP) files. (4) For quality management, annual external audit and monthly internal examination were employed. In addition, images of confusing embryos were analyzed by all the staff in a weekly held discussion.

### Blastocyst Vitrification and Warming

Vitrification has been used from 2009 onwards at our center. Embryos were vitrified within 2 h after scoring. The grade of blastocyst scored before vitrification was used for statistical analysis in this study. Before vitrification, laser treatment was used to induce shrinkage of fully expanded blastocysts. The entire vitrification procedure was performed at room temperature (22–25°C). Embryos were equilibrated in 7.5% ethylene glycol and 7.5% dimethylsulphoxide (DMSO) for 5–10 min. Then embryos were brought into vitrification medium with 15% ethylene glycol, 15% DMSO, and 0.5 mol/L sucrose, and then loaded onto the surface of the Cryotop within 40–60 s. Then embryos were submerged in liquid nitrogen immediately.

Embryos were warmed on the day of transfer at room temperature (22–25°C). They were transferred to warming solution 1 (containing 1.0 mol/L sucrose) for 1 min, followed by 3 min in warming solution 2 (containing 0.5 mol/L sucrose) and then washed twice in basal medium (washing solution 1 and 2) for 5 min each. Warmed embryos were then cultured for at least 2 h before post-warming evaluation. The temperature of warming solution 1, washing solution 2, and culture media were controlled at 37°C. After warming, blastocysts were checked for survival under the inverted microscope. Most of the blastocysts showed a significant re-expansion. Re-expanded blastocysts were scored with the same grading system (Gardner and Schoolcraft’s). After post-warming evaluation, blastocysts were transferred immediately. For surviving blastocysts without significant re-expansion, transfers were performed after obtaining informed consent from patients

### Endometrial Preparation

There were four methods for endometrial preparation. (1) Natural cycle (NC): serial trans-vaginal ultrasound scans were performed till the endometrial thickness reached ≥8 mm or approximated the level in the stimulated cycle. The timing of ovulation was estimated by a combined analysis of ultrasound, the LH level and the P level. The luteal phase support was begun from the day of transfer by administering 40 mg/d of progesterone. (2) Cycle with ovulation induction: Letrozole tablets (FURUI, Hengrui, China) 5.0 mg was administrated on days 5–9, serial ultrasound scans were performed from day 12. The following course was similar to the NC method. (3) Artificial cycle (AC): Estradiol valerate tablets (PROGYNOVA, Bayer, Germany) was administrated at 2 mg/d on days 1–4, 4 mg/d on days 5–8, and 6 mg/d on days 9–12. Serial ultrasound scans were initiated from day 10 to 12. The estrogen dosage was adjusted based on the endometrial thickness. When the endometrial thickness reached ≥8 mm or approximated the level in the stimulated cycle, progesterone was used to transform the endometrium. Blastocyst transfer was performed 5–7 days after transformation. The duration of transformation was based on the day of blastocyst development. The luteal phase support was begun from the day of transfer with the administration of 40 mg/d of progesterone. (4) Down-regulation combined with AC method: a long-acting GnRH-a agent, leuprorelin acetate (BEIYI, Lizhu, China) 3.75 mg was subcutaneously administered on the second day of menstruation. Oral estrogen was administrated on the 28th day after leuprorelin injection. The following course was similar to the AC method.

### Outcome Measures

The primary outcome measure was the live birth rate (LBR). A live birth was defined as a successful delivery of a live baby after 28 gestational weeks (22). Secondary outcome measures included clinical pregnancy rate (CPR), miscarriage rate, ectopic pregnancy rate, and monozygotic twin pregnancy. A clinical pregnancy was defined as a positive serum β-hCG 14 days after transfer, and a gestational sac can be documented by ultrasound 28–35 days after transfer. The miscarriage rate was defined as the number of clinical pregnancy losses divided by the number of clinical pregnancies (23). There were 27 patients of vanishing twin syndrome (VTS) in this study. VTS is a phenomenon of losing one of the two monozygotic embryos/fetuses, resulting in a singleton delivery. In this study, VTS was not considered as a clinical pregnancy loss. Therefore, they were counted only as live births.

### Statistical Analysis

SAS 9.2 (SAS Inc., Cary, USA) was used for statistical analysis. Numeric data were given as mean ± SD. Categorical variables were presented as number (percentage). In univariate analyses, one-way analysis of variance (ANOVA) and chi-square test were performed appropriately. Multiple logistic regression models were used to adjust for confounding factors, and to evaluate the association between each morphological parameters and pregnancy outcomes. A P value <0.05 was considered as statistically significant.

## Results

**Figure 1:**
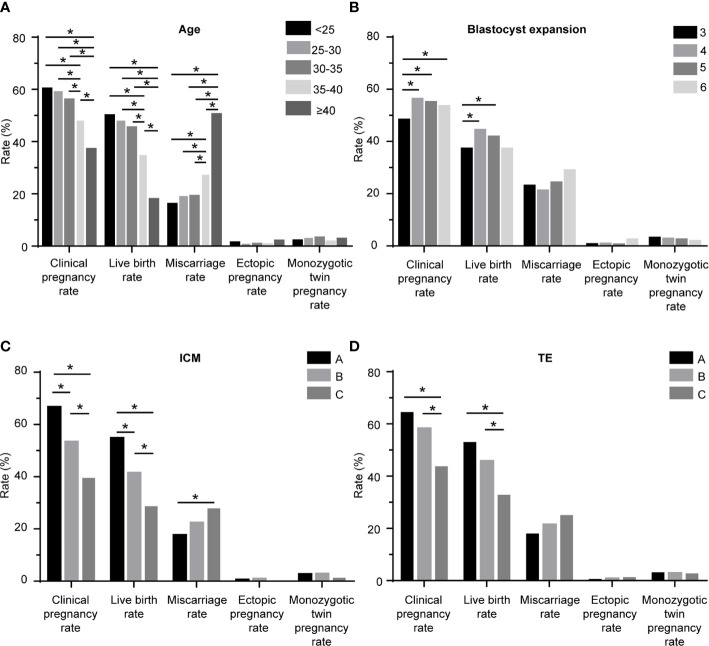
Pregnancy outcomes of the patients. **(A)** according to the age; **(B)** according to the blastocyst expansion stage; **(C)** according to the ICM grade; **(D)** according to the TE grade. ICM: inner cell mass; TE: trophectoderm; *: significant different.

A total of 10,482 SET cycles were performed, resulting in 5,680 (54.19%) clinical pregnancies and 4,429 (42.25%) live birth deliveries. The demographic and clinical features are shown in **Table 1**, according to the live birth. There were significant differences in the age at transfer, type of infertility, IVF indications, AFC, method of down-regulation, serum E2 on the day of hCG, endometrial thickness at transfer, number of oocytes retrieved, day of blastocyst development, elective transfer or not, blastocyst expansion stage, grade of ICM morphology, and grade of TE morphology between the cycles which achieved a live birth and those did not. The BMI, duration of infertility, basal FSH, fertilization method, and endometrium preparation were comparable between the two groups. **Figure 1A** shows the pregnancy outcomes according to the age at transfer. The LBR in the patients with the age of 35–40 (34.83%) and age of >40 (17.83%) were lower than those with the age of <25, 25–30, and 30–35 (49.88, 47.76, and 45.29%, respectively). Similarly, the patients with the age of 35–40 (48.04%) and >40 (37.05%) had lower CPR than those with the age of <25, 25–30, and 30–35 (60.19, 59.02, and 55.98%, respectively). On the contrary, the miscarriage rates were higher in the patients with the age of 35–40 (27.29%) and >40 (50.38%), in comparison to those with the age of <25, 25–30, and 30–35 (15.94, 18.88, 19.05%, respectively).

**Table 1:** Demographic and clinical features according to the live birth.

	Live birth n = 4,429	No live birth n = 6,053	P value
Age at transfer (year)	30.92 ± 4.21	32.46 ± 4.98	<0.001
Distribution of age, n%			<0.001
<25	208 (4.70)	209 (3.45)	
25–30	1,543 (34.84)	1,688 (27.89)	
30–35	1,813 (40.93)	2,190 (36.18)	
35–40	737 (16.64)	1,376 (22.73)	
>40	128 (2.89)	590 (9.75)	
BMI (kg/m^2^)	21.32 ± 2.79	21.79 ± 3.01	0.079
Type of infertility, n (%)			0.003
Primary infertility	2,016 (45.52)	2,934 (48.47)	
Secondary infertility	2,413 (54.48)	3,119 (51.53)	
Duration of infertility (year)	4.19 ± 3.07	4.74 ± 3.52	0.456
IVF indications, n (%)			0.040
Tubal factors	2,520 (56.90)	3,462 (57.19)	
Endometriosis	319 (7.20)	454 (7.50)	
Male factors	580 (13.10)	817 (13.50)	
Ovulation disorders	523 (11.81)	660 (10.90)	
Unexplained factors	297 (6.71)	339 (5.60)	
Diminished ovarian reserve	57 (1.29)	91 (1.50)	
Other factors	133 (3.00)	230 (3.80)	
FSH (mIU/ml)	6.61 ± 1.83	6.86 ± 2.16	0.087
AFC	15.56 ± 6.55	13.60 ± 6.74	<0.001
Down-regulation method, n (%)			<0.001
Depot GnRH-agonist	1,971 (44.50)	2,814 (46.49)	
Daily GnRH-agonist	1,772 (40.01)	2,119 (35.01)	
GnRH-antagonist	686 (15.49)	1,120 (18.50)	
Serum E2 level on the day of hCG (pg/ml)	5,566.24 ± 3,017.05	4,616.00 ± 3,082.26	<0.001
Endometrial thickness at transfer (mm)	9.47 ± 1.55	9.20 ± 1.57	0.006
No. of oocytes retrieved	15.70 ± 7.33	12.61 ± 6.46	<0.001
Fertilization method, n (%)			0.116
IVF	3,045 (68.75)	4,248 (70.18)	
ICSI	1,384 (31.25)	1,805 (29.82)	
Endometrial preparation, n (%)			0.352
NC	283 (6.39)	439 (7.25)	
AC	3,821 (86.27)	5,158 (85.21)	
DR+AC	302 (6.82)	422 (6.97)	
Ovulation induction	23 (0.52)	34 (0.56)	
Day of blastocyst development, n, (%)			<0.001
day 5	2,862 (64.62)	3,331 (55.03)	
day 6	1,543 (34.84)	2,669 (44.09)	
day 7	24 (0.54)	53 (0.88)	
Elective transfer, n, (%)	2,614 (59.02)	3,085 (50.97)	<0.001
Expansion stage, n (%)			<0.001
3	883 (19.94)	1,499 (24.76)	
4	3,230 (72.93)	4,076 (67.34)	
5	195 (4.40)	273 (4.51)	
6	121 (2.73)	205 (3.39)	
ICM grade, n (%)			<0.001
A	538 (12.15)	447 (7.38)	
B	3,823 (86.32)	5,435 (89.79)	
C	68 (1.54)	171 (2.83)	
TE grade, n (%)			<0.001
A	453 (10.23)	406 (6.71)	
B	2,939 (66.36)	3,500 (57.82)	
C	1,037 (23.41)	2,147 (35.47)	

BMI, body mass index; IVF, in vitro fertilization; FSH, follicle stimulating hormone; AFC, antral follicle count; E2, estradiol; ICSI, intracytoplasmic sperm injection; NC, natural cycle; AC, artificial cycle; DR, down-regulation; ICM, inner cell mass; TE, trophectoderm.

**Table S2** shows the LBR of blastocyst with different composite morphology of ICM and TE. When compared with blastocysts of BB (45.64%), blastocysts of AA (55.21%) and AB (53.80%) had higher LBR, while blastocysts of BC (32.37%), CB (27.70%), and CC (21.43%) had lower LBR. The LBR of blastocysts of AC (58.06%), BA (49.59%), and CA (50.00%) were similar to blastocysts of BB.

**Table 2** and **Figure 1B** show the pregnancy outcomes according to the blastocyst expansion stage. The CPR was lower in the blastocysts of stage 3 (48.15%), compared with those of stage 4 (56.15%, P = 0.015), stage 5 (54.91%, P = 0.022), and stage 6 (53.37%, P = 0.036). The LBR was lower in the blastocysts of stage 3 (37.07%), compared with those of stage 4 (44.21%, P = 0.011) and stage 5 (41.67%, P = 0.029). The rates of miscarriage, ectopic pregnancy, monozygotic twin pregnancy were comparable among the four groups.

**Table 2:** Pregnancy outcomes according to the blastocyst expansion stage.

	3n = 2,382	4n = 7,306	5n = 468	6n = 326
CPR, n (%)	1,147 (48.15)	4,102 (56.15)	257 (54.91)	174 (53.37)
OR (95%CL)	–	1.379 (1.256–1.512)	1.312 (1.075–1.600)	1.233 (0.977–1.554)
OR*^a^* (95%CL)	–	1.419 (1.278–1.575)	1.475 (1.165–1.868)	1.457 (1.099–1.932)
LBR, n (%)	883 (37.07)	3,230 (44.21)	195 (41.67)	121 (37.12)
OR (95%CL)	–	1.345 (1.223–1.480)	1.213 (0.991–1.484)	1.002 (0.789–1.273)
OR*^a^* (95%CL)	–	1.368 (1.228–1.523)	1.324 (1.041–1.684)	1.152 (0.862–1.540)
Miscarriage rate				
among CP, n (%)	258 (22.49)	843 (20.55)	61 (23.74)	49 (28.16)
OR (95%CL)	–	0.891 (0.761–1.044)	1.072 (0.780–1.475)	1.3507 (0.944–1.933)
OR*^a^* (95%CL)	–	0.903 (0.759–1.075)	1.038 (0.722–1.492)	1.256 (0.824–1.914)
Ectopic pregnancy rate				
among CP, n (%)	6 (0.52)	29 (0.71)	1 (0.39)	4 (2.30)
OR (95%CL)	–	1.354 (0.561–3.269)	0.743 (0.089–6.197)	4.475 (1.250–16.019)
OR^a^ (95%CL)	–	1.336 (0.541–3.297)	0.762 (0.089–6.543)	3.395 (0.784–14.703)
Monozygotic twin pregnancy rate				
among CP, n, (%)	34 (2.96)	109 (2.66)	6 (2.33)	3 (1.72)
OR (95%CL)	–	0.894 (0.605–1.321)	0.783 (0.325–1.884)	0.574 (0.175–1.890)
OR*^a^* (95%CL)	–	0.919 (0.603–1.400)	0.877 (0.330–2.333)	0.531 (0.123–2.301)

CPR, clinical pregnancy rate; LBR, live birth rate.

a adjustment with multiple logistic regression (covariates: age, type of infertility, IVF indications, antral follicles count, endometrial thickness, day of blastocyst development, elective transfer or not, expansion stage, ICM, and TE).

The pregnancy outcomes according to the grade of ICM are shown in **Table 3** and **Figure 1C**. The CPR (A: 66.60%, B: 53.25%, C: 39.33%; A *vs.* B: P < 0.001, A *vs.* C: P < 0.001, and B *vs.* C: P < 0.001) and LBR (A: 54.62%, B: 41.29%, C: 28.45%; A *vs.* B: P < 0.001, A *vs.* C: P < 0.001, and B *vs.* C: P < 0.001) were both decreased with decreasing grade of ICM morphology, and these differences were pairwise significant. The miscarriage rate of blastocysts with ICM grade A was lower than ICM grade C (17.53 *vs.* 27.66%, P = 0.023). No differences were found in rates of ectopic pregnancy and monozygotic twin pregnancy.

**Table 3:** Pregnancy outcomes according to the ICM grade.

	An = 985	Bn = 9,258	Cn = 239
CPR, n (%)	656 (66.60)	4,930 (53.25)	94 (39.33)
OR (95%CL)	3.076 (2.298–4.116)	1.757 (1.351–2.285)	–
OR*^a^* (95%CL)	2.539 (1.664–3.875)	1.882 (1.282–2.763)	–
LBR, n (%)	538 (54.62)	3,823 (41.29)	68 (28.45)
OR (95%CL)	3.027 (2.225–4.117)	1.769 (1.332–2.350)	–
OR*^a^* (95%CL)	2.665 (1.694–4.191)	1.941 (1.275–2.955)	–
Miscarriage rate among CP, n (%)	115 (17.53)	1070 (21.70)0.725	26 (27.66)
OR (95%CL)	0.556 (0.339–0.912)	(0.459–1.145)	–
OR*^a^* (95%CL)	0.478 (0.241–0.950)	0.633 (0.336–1.190)	–
Ectopic pregnancy rate among CP, n (%)	3 (0.46)	37 (0.75)	0 (0.00)
OR (95%CL)	–	–	–
OR*^a^* (95%CL)	–	–	–
Monozygotic twin pregnancy rate among CP, n (%)	17 (2.59)	134 (2.72)	1 (1.06)
OR (95%CL)	2.474 (0.325–18.810)	2.598 (0.360–18.780)	–
OR*^a^* (95%CL)	1.160 (0.141–9.520)	1.349 (0.181–10.042)	–

ICM, inner cell mass; CPR, clinical pregnancy rate; LBR, live birth rate.

a adjustment with multiple logistic regression (covariates: age, type of infertility, IVF indications, antral follicles count, endometrial thickness, day of blastocyst development, elective transfer or not, expansion stage, ICM, and TE).

**Table 4** and **Figure 1D** illustrate the pregnancy outcomes as a function of TE. Blastocysts with TE morphology of C had a lower CPR (C: 43.53%, A: 64.26%, B: 58.11%; C *vs.* A: P = 0.003, C *vs.* B: P < 0.001) and a lower LBR (C: 32.57%, A: 52.74%, B: 45.64%; C *vs.* A: P < 0.001, C *vs.* B: P < 0.001), compared with those with TE morphology of A and B. The CPR and LBR were comparable between the group of TE grade A and B. No differences were found in rates of miscarriage, ectopic pregnancy, and monozygotic twin pregnancy. The details of the multiple logistics regression model for live birth are shown in **Table 5**.

**Table 4:** Pregnancy outcomes according to the TE grade.

	An = 859	Bn = 6,439	Cn = 3,184
CPR, n (%)	552 (64.26)	3,742 (58.11)	1,386 (43.53)
OR (95%CL)	2.333 (2.000–2.727)	1.800 (1.652–1.961)	–
OR*^a^* (95%CL)	1.728 (1.391–2.147)	1.737 (1.575–1.915)	–
LBR, n (%)	453 (52.74)	2,939 (45.64)	1,037 (32.57)
OR (95%CL)	2.310 (1.982–2.692)	1.739 (1.591–1.900)	–
OR*^a^* (95%CL)	1.698 (1.372–2.101)	1.651 (1.492–1.827)	–
Miscarriage rate among CP, n (%)	97 (17.57)	779 (20.82)0.825	335 (24.17)
OR (95%CL)	0.669 (0.520–0.860)	(0.713–0.955)	–
OR*^a^* (95%CL)	0.802 (0.570–1.128)	0.864 (0.734–1.017)	
Ectopic pregnancy rate among CP, n (%)	2 (0.36)	24 (0.64)	14 (1.01)
OR (95%CL)	0.356 (0.081–1.573)	0.633 (0.326–1.226)	–
OR*^a^* (95%CL)	0.547 (0.100–2.991)	0.774 (0.375–1.601)	
Monozygotic twin pregnancy rate among CP, n, (%)	16 (2.90)	101 (2.70)	35 (2.53)
OR (95%CL)	1.152 (0.632–2.099)	1.071 (0.725–1.581)	–
OR*^a^* (95%CL)	1.091 (0.481–2.473)	1.071 (0.703–1.634)	–

TE, trophectoderm; CPR, clinical pregnancy rate; LBR, live birth rate.

a adjustment with multiple logistic regression (covariates: age, type of infertility, IVF indications, antral follicles count, endometrial thickness, day of blastocyst development, elective transfer or not, expansion stage, ICM, and TE).

**Table 5:** The results of the multiple logistics regression model for live birth.

	β	S.E.	Waldχ2	P value	OR	95%CL
Age	−0.0701	0.0049	201.6450	<0.0001	0.932	0.923–0.941
Type of infertility						
Primary infertility	ref	–	–	–	–	–
Secondary infertility	−0.0545	0.0317	2.9614	0.0853	0.897	0.792–1.015
IVF indications						
Tubal factors	ref	–	–	–	–	–
Endometriosis	0.0775	0.2384	0.1057	0.7451	0.951	0.666–1.356
Male factors	−0.0699	0.2482	0.0792	0.7783	0.820	0.542–1.242
Ovulation disorders	0.4559	0.2468	3.4130	0.0647	1.388	0.945–2.037
Unexplained factors	0.1901	0.2399	0.6279	0.4281	1.064	0.734–1.543
Diminished ovarian reserve	−0.7819	0.9188	0.7226	0.3953	0.403	0.046–3.504
Other factors	0.1282	0.2029	0.3993	0.5274	0.721	0.491–1.058
AFC	0.0270	0.00529	26.1073	<0.0001	1.027	1.017–1.038
Endometrial thickness	0.0993	0.0145	46.7564	<0.0001	1.104	1.073–1.136
Day of development						
Day 5	ref	–	–	–	–	–
Day 6	−0.0424	0.0955	0.1966	0.0066	0.753	0.684–0.828
Day 7	−0.1995	0.1849	1.1647	0.2805	0.643	0.373–1.110
Elective transfer or not						
Elective	0.1360	0.0439	9.5867	0.0020	1.313	1.105–1.559
Non-elective	ref	–	–	–	–	–
Expansion sage						
3	ref	–	–	–	–	–
4	0.1292	0.0505	6.5486	0.0105	1.368	1.228–1.523
5	0.0969	0.0914	1.1247	0.0289	1.324	1.041–1.684
6	−0.0422	0.1085	0.1516	0.6970	1.152	0.862–1.540
ICM						
A	0.4323	0.0930	21.6264	<0.0001	2.665	1.694–4.191
B	0.1153	0.0786	2.1524	<0.0001	1.941	1.275–2.955
C	ref	–	–	–	–	–
TE						
A	0.1859	0.0679	7.4946	<0.0001	1.698	1.372–2.101
B	0.1577	0.0396	15.8901	<0.0001	1.651	1.492–1.827
C	ref	–	–	–	–	–

## Discussion

In this study, we evaluated the association between the three morphology parameters of blastocyst and pregnancy outcomes. We found that the expansion stage, ICM grade, and TE grade were all independent predictors of live birth and clinical pregnancy. The ICM grade was the strongest characteristic in predicting live birth.

The scoring system according to morphology is the most commonly used method for human blastocyst assessment (21). SET serves as an ideal model for analyzing the predicting value of each morphology parameter on pregnancy outcomes. Previous studies have indicated that the expansion stage predicts clinical pregnancy and/or live birth (24–26). Our study is generally in agreement with them. The CPR and LBR were both lower in blastocysts of stage 3 than those of stages 4–5. There are extensive morphology change and energy utilization during the expansion and hatching of a blastocyst (27, 28). Therefore, the expansion stage reflects the viability of a blastocyst. TE has also been reported as an effective predictor of pregnancy outcomes (24, 25). We found a lower CPR and a lower LBR in blastocysts with TE grade C, compared with those with A and B. TE is destined to become the placenta. TE grade is a reflection of the potential to attach, invade, and implant into the endometrium. Therefore, it is not surprising that TE grade predicts the probability of pregnancy and live birth. In addition, TE morphology is possibly associated with blastocyst aneuploidy and mosaicism (29).

There is a continuing debate over the most important morphological characteristic in embryo assessment. Previous studies suggested that TE morphology and embryo stage were better predictors of live birth (20, 25), but our study indicates that ICM grade has the most significant predicting effect. From the aspect of statistical calculation, when the live birth was set as the dependent variable, the multiple logistic regression showed that the absolute values of standardized estimate of ICM grades A and B were 0.0169 and 0.0325, respectively. They are larger than those of TE grade A and C (0.0069 and 0.0083, respectively). Therefore, ICM has a stronger effect on the outcome of the model than TE (**Table S1**). From the aspect of clinical application, the LBR increased with the increasing of the score of ICM (A: 54.62%, B: 41.29%, C: 28.45%), and these differences were pairwise significant. The blastocysts with TE grade C (32.57%) yielded a lower LBR, compared with those of TE grade A and B, but there was no difference in LBR between the blastocysts of TE grade A (52.74%) and B (45.64%). The ICM is destined to become the fetus, and therefore reflects the viability of the blastocyst. Surprisingly, many studies found that ICM was not associated with the likelihood of pregnancy or live birth (20, 30). The rationale of this discrepancy is possibly attributed to methodological heterogeneity among studies, especially in study population, clinical and laboratory practice, and analysis method. Firstly, there are significant differences in distribution of blastocysts with different morphology grades. Some studies were of small sample size, and included very few or even no blastocysts with ICM grade C. Therefore, the effect of ICM grade may be underestimated in these studies. Secondly, some studies employed fresh embryo transfer. Several factors may confound their results, such as ovarian stimulation, altered implantation window by supra-physiologic hormone level. Thirdly, the culture conditions were different among studies, such as culture media, incubator, and gas tension. Vitrolife Sequential was the most popularly used culture media, while other culture media were also used, such as Cook Sequential and Quinn’s advantage, with or without supplement. A recent large study found an impact of culture medium on pregnancy outcomes (31). There was also a significant difference in the type of incubator among studies. In addition, some studies did not provide the details of their culture conditions. The laboratory techniques have been significantly updated in recent years. The results from old studies, such as those using slow freezing, might be less meaningful. Some studies used multiple blastocysts transfer. Obviously, results based on SET are more reliable. Finally, several centers performed modifications to the Gardner and Schoolcraft’s evaluation system according to their own conditions and habits. Together with the present study, the predicting value of ICM was confirmed by more recent studies with large sample size and sufficient number of blastocysts with ICM grade C (15, 17, 32). ICM also reflects the potential to maintain the pregnancy. Van den Abbeel et al. found a lower risk of pregnancy loss in blastocysts with ICM grade A (33). Shi et al. reported that ICM grade predicted the risk of miscarriage in both euploidy and aneuploidy blastocysts (34). Similarly, we found a higher risk of miscarriage in blastocysts with ICM grade C compared to those with ICM grade A.

Besides morphological parameters, several factors may also have an impact on pregnancy outcomes. Firstly, previous studies showed that ICSI resulted in similar or lower CPR and LBR in patients with normal sperm, compared with IVF (35, 36). Of note, these results were from fresh transfer cycles. Our result indicates that fertilization method has no significant effect on pregnancy outcomes in frozen transfer cycles. An ongoing RCT is being performed to compare the efficacy of ICSI and IVF. Its primary endpoint is live birth of the first embryo transfer after IVF/ICSI, including both fresh and frozen transfer (37). Secondly, our previous study showed the endometrial preparation protocol of AC had a higher LBR, compared with NC protocol (38). Since the advantages in success rate and treatment programming, we used AC method in the most of FET cycles. In this study, the type of endometrial preparation had no significant effect on pregnancy outcomes. The rationale is that most of the cycles employed AC protocol (85.7%). Thirdly, previous studies have demonstrated a higher LBR after transfer with day 5 blastocyst, compared with day 6 blastocyst. Embryos reaching blastocyst stage on day 7 or 8 are of delayed development, and have worse prognosis for implantation and pregnancy (13). Our data is in line with this result. In this study, 59.1% of blastocysts were vitrified on day 5, 40.2% of them were vitrified on day 6, and only 0.7% of them were vitrified on day 7. The day of blastocyst development was an independent predictor of live birth.

The implication for practice of this study is that it provides a non-invasive, convenient, economic, and effective embryo selection strategy for SET. Based on the results of this study, we propose that a blastocyst with stage 4–5, ICM grade A, and TE grade A/B should be given priority for SET. In addition, a blastocyst with ICM grade B is also acceptable. Our results can also help clinicians in patient counseling. Both doctors and patients often face the dilemma that a low-quality blastocyst should be used or discarded. The present study suggests that careful explanation should be provided before transfer with a blastocyst with stage 3, ICM morphology of C, or TE morphology of C, because of its limited potential for pregnancy and live birth. Blastocysts with ICM grade C had a higher risk of miscarriage. Therefore, for patients planning to use this type of blastocysts, clinicians should inform them in details about the risks, especially for patients with inherent potential for pregnancy loss (e.g. recurrent spontaneous abortion, uterine malformation, and cervical insufficiency) and patients who are more vulnerable to miscarriage (e.g. intrauterine adhesion and mental disorder).

The implication for research is that this study suggests a need for studies on SET with PGT-A on this issue. PGT-A is a novel technique, which can help embryo selection. However, the clinical data from SET with PGT-A is rare. Boynukalin et al. found a higher LBR in blastocysts with ICM grade of A or B (17). Of note, the patients of their study are not general population. Their indications for PGT-A were advanced maternal age, repeated implantation failure, recurrent miscarriage, and combined indications. Nazem et al. found a higher likelihood of ongoing pregnancy/live birth in blastocysts with ICM grade of A, and TE grade of A or B, which is similar to our finding (32). Shi et al. examined the chorionic villi of miscarried conceptuses, and found that ICM grade was associated with the occurrence of aneuploidy. Their sample size is small (91 cases of euploidy and 83 cases of aneuploidy) (34). Since PGT-A is not performed in patients with normal chromosomal karyotype at our center, the cycles analyzed in this study is all without PGT-A. However, the result was generally in agreement with previous studies with PGT-A, highlighting the importance of ICM grade. The possible rationale is that the large sample size of this study and adjustment with multiple regression analysis reduce the impact of aneuploidy/mosaic on pregnancy outcomes.

The strength of this study is its large sample size. Over 10,000 SET cycles were analyzed. There were several limitations in this study. Firstly, selection bias and confounders may affect the result, because of the retrospective nature; nevertheless, multivariate logistic regression was used to adjust for their potential effects. Secondly, there were some modifications during the inclusion period, such as culture media, incubators, and gas tension; however, the general culture conditions were stable, as monitored by our quality management. Thirdly, morphological grading is a subjective tool, where interpersonal variation may contribute to the outcome; nevertheless, measures were performed to maintain standardized scoring. Only the grades scored before vitrification were used for statistical analysis in this study. Additionally, the numbers of blastocysts with ICM/TE morphology of C are relatively low, resulting in unbalanced subgroups. Finally, the cycles were without PGT-A. The effect of embryo aneuploidy and mosaicism on pregnancy outcomes cannot be ruled out. A further prospective study with PGT-A can be performed to confirm our findings.

In conclusion, blastocyst expansion stage, ICM grade, and TE grade are all associated with pregnancy outcomes after SET. ICM grade is the strongest predictor of live birth. A blastocyst with stage 4–5, ICM grade A, and TE grade A/B should be given priority for SET.

## Data Availability Statement

The original contributions presented in the study are included in the article/**Supplementary Material**. Further inquiries can be directed to the corresponding author.

## Ethics Statement

The studies involving human participants were reviewed and approved by the Institution Review Board (IRB) of Tongji Hospital. The patients/participants provided their written informed consent to participate in this study.

## Author Contributions

All authors contributed to the study conception and design. JA, LJ, YZ, and PY performed data collection and analysis. JA, BH, and XD performed data interpretation. JA and LJ drafted the first manuscript, and all authors reviewed and gave substantial revisions. All authors contributed to the article and approved the submitted version.

## Funding

This work was supported by the National Natural Science Foundation of China (81801531).

## Conflict of Interest

The authors declare that the research was conducted in the absence of any commercial or financial relationships that could be construed as a potential conflict of interest.

## References

[1] Practice Committee of American Society for Reproductive Medicine. Multiple gestation associated with infertility therapy: an American Society for Reproductive Medicine Practice Committee opinion. Fertil Steril (2012) 97:4. 10.1016/j.fertnstert.2011.11.048 22192352

[2] Santana DS Cecatti JG Surita FG Silveira C Costa ML Souza JP. Twin Pregnancy and Severe Maternal Outcomes: The World Health Organization Multicountry Survey on Maternal and Newborn Health. Obstetr Gynecol (2016) 127:4. 10.1097/AOG.0000000000001338 26959199

[3] Practice Committee of the American Society for Reproductive Medicine. Guidance on the limits to the number of embryos to transfer: a committee opinion. Fertil Steril (2017) 107:4.28040094

[4] Rienzi L Gracia C Maggiulli R LaBarbera AR Kaser DJ Ubaldi FM. Oocyte, embryo and blastocyst cryopreservation in ART: systematic review and meta-analysis comparing slow-freezing versus vitrification to produce evidence for the development of global guidance. Hum Reprod Update (2017) 23:2. 10.1093/humupd/dmw038 PMC5850862 27827818

[5] Shi Y Sun Y Hao C Zhang H Wei D Zhang Y. Transfer of Fresh versus Frozen Embryos in Ovulatory Women. N Engl J Med (2018) 378:2. 10.1056/NEJMoa1705334 29320646

[6] Yang M Lin L Sha C Li T Gao W Chen L. Which is better for mothers and babies: fresh or frozen-thawed blastocyst transfer? BMC Pregnancy Childbirth (2020) 20:1. 10.1186/s12884-020-03248-5 PMC7513314 32967652

[7] Stormlund S Sopa N Zedeler A Bogstad J Prætorius L Nielsen HS. Freeze-all versus fresh blastocyst transfer strategy during in vitro fertilisation in women with regular menstrual cycles: multicentre randomised controlled trial. BMJ (2020) 370:m2519. 32759285 10.1136/bmj.m2519 PMC7399608

[8] El-Toukhy T Wharf E Walavalkar R Singh A Bolton V Khalaf Y. Delayed blastocyst development does not influence the outcome of frozen-thawed transfer cycles. BJOG An Int J Obstetr Gynaecol (2011) 118:13. 10.1111/j.1471-0528.2011.03101.x 21895955

[9] Sunkara SK Siozos A Bolton VN Khalaf Y Braude PR El-Toukhy T. The influence of delayed blastocyst formation on the outcome of frozen-thawed blastocyst transfer: a systematic review and meta-analysis. Hum Reprod (2010) 25:8. 10.1093/humrep/deq143 20542896

[10] Bourdon M Pocate-Cheriet K Finet de Bantel A Grzegorczyk-Martin V Amar Hoffet A Arbo E. Day 5 versus Day 6 blastocyst transfers: a systematic review and meta-analysis of clinical outcomes. Hum Reprod (2019) 34:10. 10.1093/humrep/dez163 PMC7967799 31644803

[11] Tubbing A Shaw-Jackson C Ameye L Colin J Rozenberg S Autin C. Increased live births after day 5 versus day 6 transfers of vitrified-warmed blastocysts. J Assisted Reprod Genet (2018) 35:3. 10.1007/s10815-017-1097-x PMC5904067 29204868

[12] Kovalevsky G Carney SM Morrison LS Boylan CF Neithardt AB Feinberg RF. Should embryos developing to blastocysts on day 7 be cryopreserved and transferred: an analysis of pregnancy and implantation rates. Fertil Steril (2013) 100:4. 10.1016/j.fertnstert.2013.06.021 23876530

[13] Alpha Scientists in Reproductive Medicine and ESHRE Special Interest Group of Embryology. The Istanbul consensus workshop on embryo assessment: proceedings of an expert meeting. Hum Reprod (2011) 26:6. 10.1093/humrep/der037 21502182

[14] Gardner DK Lane M Stevens J Schlenker T Schoolcraft WB. Blastocyst score affects implantation and pregnancy outcome: towards a single blastocyst transfer. Fertil Steril (2000) 73:6. 10.1016/s0015-0282(00)00518-5 10856474

[15] Subira J Craig J Turner K Bevan A Ohuma E McVeigh E. Grade of the inner cell mass, but not trophectoderm, predicts live birth in fresh blastocyst single transfers. Hum Fertil (2016) 19:4. 10.1080/14647273.2016.1223357 27624529

[16] Richter KS Harris DC Daneshmand ST Shapiro BS. Quantitative grading of a human blastocyst: optimal inner cell mass size and shape. Fertil Steril (2001) 76:6. 10.1016/s0015-0282(01)02870-9 11730744

[17] Boynukalin FK Gultomruk M Cavkaytar S Turgut E Findikli N Serdarogullari M. Parameters impacting the live birth rate per transfer after frozen single euploid blastocyst transfer. PLoS One (2020) 15:1. 10.1371/journal.pone.0227619 PMC6957140 31929583

[18] Honnma H Baba T Sasaki M Hashiba Y Ohno H Fukunaga T. Trophectoderm morphology significantly affects the rates of ongoing pregnancy and miscarriage in frozen-thawed single-blastocyst transfer cycle in vitro fertilization. Fertil Steril (2012) 98:2. 10.1016/j.fertnstert.2012.05.014 22682029

[19] Ahlstrom A Westin C Reismer E Wikland M Hardarson T. Trophectoderm morphology: an important parameter for predicting live birth after single blastocyst transfer. Hum Reprod (2011) 26:12. 10.1093/humrep/der325 21972253

[20] Chen X Zhang J Wu X Cao S Zhou L Wang Y. Trophectoderm morphology predicts outcomes of pregnancy in vitrified-warmed single-blastocyst transfer cycle in a Chinese population. J Assisted Reprod Genet (2014) 31:11. 10.1007/s10815-014-0317-x PMC4389933 25123128

[21] Schoolcraft WB Gardner DK Lane M Schlenker T Hamilton F Meldrum DR. Blastocyst culture and transfer: analysis of results and parameters affecting outcome in two in vitro fertilization programs. Fertil Steril (1999) 72:4. 10.1016/s0015-0282(99)00311-8 10521095

[22] Zegers-Hochschild F Adamson GD de Mouzon J Ishihara O Mansour R Nygren K. The International Committee for Monitoring Assisted Reproductive Technology (ICMART) and the World Health Organization (WHO) Revised Glossary on ART Terminology, 2009. Hum Reprod (2009) 24:11. 10.1093/humrep/dep343 19801627

[23] Li J Yin M Wang B Lin J Chen Q Wang N. The effect of storage time after vitrification on pregnancy and neonatal outcomes among 24 698 patients following the first embryo transfer cycles. Hum Reprod (2020) 35:7. 10.1093/humrep/deaa136 32575120

[24] Bakkensen JB Brady P Carusi D Romanski P Thomas AM Racowsky C. Association between blastocyst morphology and pregnancy and perinatal outcomes following fresh and cryopreserved embryo transfer. J Assisted Reprod Genet (2019) 36:11. 10.1007/s10815-019-01580-0 PMC6885471 31512049

[25] Thompson SM Onwubalili N Brown K Jindal SK McGovern PG. Blastocyst expansion score and trophectoderm morphology strongly predict successful clinical pregnancy and live birth following elective single embryo blastocyst transfer (eSET): a national study. J Assisted Reprod Genet (2013) 30:12. 10.1007/s10815-013-0100-4 PMC3843172 24114628

[26] Zhao J Yan Y Huang X Sun L Li Y. Blastocoele expansion: an important parameter for predicting clinical success pregnancy after frozen-warmed blastocysts transfer. Reprod Biol Endocrinol RB&E (2019) 17:1. 30674332 10.1186/s12958-019-0454-2 PMC6344998

[27] Wrenzycki C Herrmann D Niemann H. Timing of blastocyst expansion affects spatial messenger RNA expression patterns of genes in bovine blastocysts produced in vitro. Biol Reprod (2003) 68:6. 10.1095/biolreprod.102.012104 12606328

[28] Guo J Kim NH Cui XS. Inhibition of Fatty Acid Synthase Reduces Blastocyst Hatching through Regulation of the AKT Pathway in Pigs. PLoS One (2017) 12:1. 10.1371/journal.pone.0170624 PMC5249155 28107461

[29] Alfarawati S Fragouli E Colls P Stevens J Gutierrez-Mateo C Schoolcraft WB. The relationship between blastocyst morphology, chromosomal abnormality, and embryo gender. Fertil Steril (2011) 95:2. 10.1016/j.fertnstert.2010.04.003 20537630

[30] Du QY Wang EY Huang Y Guo XY Xiong YJ Yu YP. Blastocoele expansion degree predicts live birth after single blastocyst transfer for fresh and vitrified/warmed single blastocyst transfer cycles. Fertil Steril (2016) 105:4. 26776910 10.1016/j.fertnstert.2015.12.014

[31] Castillo CM Harper J Roberts SA O’Neill HC Johnstone ED Brison DR. The impact of selected embryo culture conditions on ART treatment cycle outcomes: a UK national study. Hum Reprod Open (2020) 2020:1. 10.1093/hropen/hoz031 PMC7016773 32083189

[32] Nazem TG Sekhon L Lee JA Overbey J Pan S Duke M. The correlation between morphology and implantation of euploid human blastocysts. Reprod Biomed Online (2019) 38:2. 10.1016/j.rbmo.2018.10.007 30579820

[33] Van den Abbeel E Balaban B Ziebe S Lundin K Cuesta MJ Klein BM. Association between blastocyst morphology and outcome of single-blastocyst transfer. Reprod Biomed Online (2013) 27:4. 23953585 10.1016/j.rbmo.2013.07.006

[34] Shi D Xu J Zhang M Niu W Shi H Yao G. Association between the quality of inner cell mass and first trimester miscarriage after single blastocyst transfer. Reprod Biol Endocrinol RB&E (2020) 18:1. 32398002 10.1186/s12958-020-00595-y PMC7216576

[35] Boulet SL Mehta A Kissin DM Warner L Kawwass JF Jamieson DJ. Trends in use of and reproductive outcomes associated with intracytoplasmic sperm injection. JAMA (2015) 313:3. 10.1001/jama.2014.17985 PMC4343214 25602996

[36] Tannus S Son WY Gilman A Younes G Shavit T Dahan MH. The role of intracytoplasmic sperm injection in non-male factor infertility in advanced maternal age. Hum Reprod (2017) 32:1. 27852688 10.1093/humrep/dew298

[37] Dang VQ Vuong LN Ho TM Ha AN Nguyen QN Truong BT. The effectiveness of ICSI versus conventional IVF in couples with non-male factor infertility: study protocol for a randomised controlled trial. Hum Reprod Open (2019) 2019:2. 10.1093/hropen/hoz006 PMC6436611 30937394

[38] Zheng Y Dong X Huang B Zhang H Ai J. The artificial cycle method improves the pregnancy outcome in frozen-thawed embryo transfer: a retrospective cohort study. Gynecol Endocrinol (2015) 31:1. 25223893 10.3109/09513590.2014.958988

