# Supplementary material for: The Morphology of Inner Cell Mass Is the Strongest Predictor of Live Birth After a Frozen-Thawed Single Embryo Transfer
Source: Front Endocrinol (Lausanne). 2021 Feb 24;12:621221. doi: 10.3389/fendo.2021.621221 (PMC7943864; doi:10.3389/fendo.2021.621221)
Supplement: Supplementary file 1 [file DataSheet_1.docx]

Table S1. The result of multiple logistic regression of live birth according to different scores of ICM and TE

| Predictors | Estimate | Standard error | Wald X2 | P value | Standardized estimate |
| --- | --- | --- | --- | --- | --- |
| ICM A | 0.4390 | 0.0700 | 39.3599 | <0.001 | 0.0169 |
| ICM C | -0.5858 | 0.1006 | 33.9102 | <0.001 | -0.0325 |
| TE A | 0.2216 | 0.0562 | 15.5759 | <0.001 | 0.0069 |
| TE C | -0.3830 | 0.0393 | 94.9763 | <0.001 | -0.0083 |

Table S2. The live birth rate according to the composite morphology of ICM and TE

| Composite morphology of ICM and TE | LBR, n, (%) | OR (95% CI) | OR^a^ (95% CI) |
| --- | --- | --- | --- |
| AA  AB  AC | 265 (55.21)  255 (53.80)  18 (58.06) | 1.4683 (1.218-1.771)  1.3871 (1.150-1.674)  1.6494 (0.807-3.373) | 1.365 (1.127-1.652)  1.315 (1.085-1.594)  1.742 (0.845-3.594) |
| BA  BB  BC | 182 (49.59)  2625 (45.64)  1016 (32.37) | 1.1719 (0.949-1.447)  -  0.5701 (0.521-0.624) | 1.083 (0.782-1.343)  -  0.573 (0.522-0.629) |
| CA  CB  CC | 6 (50.00)  59 (27.70)  3 (21.43) | 1.1912 (0.384-3.698)  0.4564 (0.337-0.619)  0.3249 (0.091-1.166) | 1.158 (0.368-3.640)  0.450 (0.331-0.613)  0.326 (0.090-1.180) |
